# Profil histologique et évolution des tumeurs intra rachidiennes opérées

**DOI:** 10.11604/pamj.2021.38.128.21214

**Published:** 2021-02-04

**Authors:** Sylvain Denléwendé Zabsonre, Augustin Tozoula Bambara, Souleymane Ouattara, Adama Traore, Adeline Julie Kyelem Kafando, Alfred Aselme Dabilgou, Stéphane Klamadji, Yacouba Haro, Ibrahim Dao, Boureima Kinda, Abel Kabre

**Affiliations:** 1 Service de Neurochirurgie du Centre Hospitalier Universitaire Yalgado Ouédraogo de Ouagadougou, Ouagadougou, Burkina Faso

**Keywords:** Tumeur, rachis, méningiome, neurinome, épendymome, Tumor, spine, meningioma, neurinoma, ependymoma

## Abstract

Les tumeurs intrarachidiennes sont peu fréquentes. Leur diagnostic positif est basé sur l´imagerie médicale surtout l´IRM. L´anatomopathologie apporte la certitude du diagnostic. La chirurgie est le traitement de choix pour la plupart d´entre elles. Le pronostic est fonction de la nature histologique et de l´état clinique initial du patient. Nous rapportons le profil histologique et l´évolution des tumeurs intra rachidiennes opérées dans notre service. Il s´agissait d´une étude rétrospective sur une période 10 ans. Ont été inclus les cas opérés confirmés à l´histologie (23 cas). Quatre dossiers non exploitables ont été exclus. Les patients ont consulté en moyenne 79 jours après les premiers symptômes pour un syndrome de compression médullaire lente dans 11 cas. Sept TDM et 14 IRM permettaient d´objectiver 4 tumeurs intramédullaires, 9 intradurales, 1 extra durale et 5 localisations non précises. L´histologie concluait à un méningiome dans 11 cas, un neurinome dans 3 cas. Elle a conclu au même diagnostic que l´imagerie médicale dans 7 cas (4 TDM et 3 IRM). L´exérèse était macroscopiquement complète dans 14 cas, partielle dans 5 cas. Après un recul de 6 mois, 6 patients avaient récupéré totalement, 9 partiellement. Le diagnostic était tardif. L´IRM décrivait mieux la lésion, mais était limitée dans l´approche histologique. Les méningiomes dominaient. L´exérèse était le plus souvent complète. Les suites opératoires étaient favorables.

## Abstract (en)

Intraspinal tumors are uncommon. Positive diagnosis is based on medical imaging exams, in particular MRI. Anatomopathological examination allows for definitive diagnosis. Surgery is the treatment of choice for most of them. Prognosis is related to the histological type and patient´s initial clinical condition. We here report the histological profile and progression of intraspinal tumors in our department. We conducted a retrospective study over a period of 10 years. All operated patients with histology-confirmed intraspinal tumor (23 cases) were enrolled. Four unworkable records were excluded. The median time from symptom onset and first consultation was 79 days. Patients presented with spinal cord compression. In 11 cases this was characterized by slow onset. Seven CT scan and 14 MRI were performed, which showed 4 intramedullary tumors, 9 intradural, 1 extradural, and 5 of unknown site. Histological examination showed meningioma in 11 cases, neurinoma in 3 cases. In 7 cases, it confirmed the diagnosis based on Imaging tests (4 CT and 3 MRI). Macroscopically complete resection was performed in 14 cases; it was partial in 5 cases. After a 6-month follow-up period 6 patients had fully recovered, 9 partially. This study highlights diagnosis delays. MRI better defined the lesion, but its histological approach was limited. Meningiomas dominated. Complete resection was most often performed. The postoperative course was uneventful.

## Introduction

Les tumeurs intrarachidiennes sont peu fréquentes parmi les tumeurs primitives du système nerveux central et de ses enveloppes [1,2]. En fonction de leur point de départ, ces tumeurs sont divisées en trois groupes: les tumeurs intramédullaires, les tumeurs intradurales extra médullaires et les tumeurs extradurales intra canalaires. Les tumeurs de la queue de cheval se développent aux dépens des structures constitutives de la queue de cheval: filum, gaines nerveuses et autres structures tissulaires intra canalaires. La grande majorité de ces tumeurs est bénigne. Le diagnostic clinique de ces tumeurs est difficile, car en dehors des signes de compression médullaire ou radiculaire, elles n´ont aucun signe clinique spécifique. L´imagerie médicale en occurrence l´imagerie par résonance magnétique (IRM) contribue très fortement au diagnostic positif précoce de ces tumeurs [3-6]. L´anatomopathologie apporte la certitude du diagnostic. Le traitement de choix pour la plupart de ces tumeurs est l´exérèse chirurgicale la plus complète possible. Ce traitement peut être associé à un traitement complémentaire en fonction de l´histologie de la tumeur. Le pronostic est fonction de la nature histologique de la tumeur et de l´état clinique initial du patient. Nous rapportons le profil histologique et l´évolution des tumeurs intra rachidiennes opérées dans notre service.

## Méthodes

Il s´agissait d´une étude rétrospective à visée descriptive portant sur une période 10 ans allant du premier janvier 2009 au 31 décembre 2018. Elle concernait les dossiers des patients admis pour un syndrome de compression médullaire et/ou radiculaire dans le service de neurochirurgie du Centre Hospitalier Universitaire Yalgado Ouédraogo de Ouagadougou (CHU-YO). Ont été inclus les patients chez qui le diagnostic de tumeur intrarachidienne a été évoqué par la clinique et la paraclinique et confirmé à l´examen anatomopathologique de la pièce opératoire. N´ont pas été inclus dans notre étude: 1) les compressions médullaires lentes non traumatiques d´autres étiologies (vasculaire, infectieuse ou arthrosiques …); 2) les tumeurs vertébrales sans extension intra canalaire; 3) les tumeurs intrarachidiennes non opérées. Ainsi 23 cas de tumeurs intrarachidiennes colligés. Quatre cas ont été exclus, car leur dossier n´était pas exploitable (les résultats de l´examen anatomopathologique n´ont pas été retrouvés). Dix-neuf 19 cas ont été inclus l´étude. L´âge moyen des patients dont le dossier a été inclus la série était de 36 ans avec des extrêmes de 12 ans et 66 ans et un écart type de 16,67. Le sex-ratio était de 2,8 (14 hommes/ 5 femmes).

## Résultats

**Données diagnostiques**: le délai moyen entre le début des symptômes et la première consultation dans notre service était de 79 jours avec des extrêmes de 21 et 180 jours et un écart type de 50,72. Le motif de consultation était une impotence fonctionnelle d´un ou de plusieurs membres dans 17 cas et une dorsalgie dans 2 cas. A l´examen physique 8 patients avaient un état général altéré et 11 un état général conservé. Une douleur rachidienne provoquée était notée dans 4 cas. Tous les patients avaient un déficit moteur (11 para parésies, 7 paraplégie et 1 tétraplégie) d´installation progressive. Ce déficit moteur était associé à un trouble sensitif dans 18 cas (3 anesthésies et 15 hypoesthésies dont 12 cas avec un niveau sensitif); des troubles sphinctériens dans 12 cas (7 cas de rétention urinaire avec constipation et 5 cas d´incontinence urinaire); des troubles des reflex ostéotendineux (ROT) dans 7 cas (4 ROT vifs et 3 ROT abolis). Il a été conclu à un syndrome de compression médullaire cervical dans 1 cas, dorsal dans 10 cas et un syndrome de la queue de cheval dans 2 cas. Dans 1 cas il s´agissait d´un syndrome d´irritation tétra pyramidal. Aucun syndrome n´a pu être retenu dans 5 cas. Une tomodensitométrie (TDM) a été réalisée dans 7 cas et une imagerie par résonance magnétique (IRM) dans 14 cas, dont 2 après réalisation de la TDM. A l´imagerie médicale (TDM, IRM), la tumeur siégeait en cervical dans 3 cas, en dorsal dans 12 cas, et en lombaire dans 4 cas. Elle était intramédullaire dans 4 cas, intradurale extra médullaire dans 9 cas, extra duraux intra canalaire dans 1 cas. Dans 5 cas (4 TDM et 1 IRM), le siège intra canalaire de la tumeur n´a pu être précisé. Au scanner, l´approche histologique était donnée dans 4 cas, dont 3 méningiomes et 1 neurinome. A l´IRM, elle était donnée dans 3 cas, dont 2 méningiomes, un épendymome. L´anatomie pathologie concluait à un méningiome dans 11 cas, un neurinome dans 3 cas. Sept diagnostics histologiques évoqués à l´imagerie médicale (4 TDM et 3 IRM) ont été confirmés par l´anatomopathologie. Le Tableau 1 présente les différents diagnostics retenus à l´imagerie et à l´anatomopathologie.

**Tableau 1:** diagnostics retenus à l´imagerie et à l´anatomopathologie

Approche histologique	Tomodensitométrie (TDM)	Résonnance infra magnétique (IRM)	Anatomopathologie
Méningiome	3	2	11
Neurinome	1	0	3
Ependymome	0	1	2
Hémangiome	0	0	1
Plasmocytome	0	0	1
ostéome ostéoide	0	0	1
Pas d´approche histologique	3	14	0
**Total**	7	21	19

**Figure 1:**
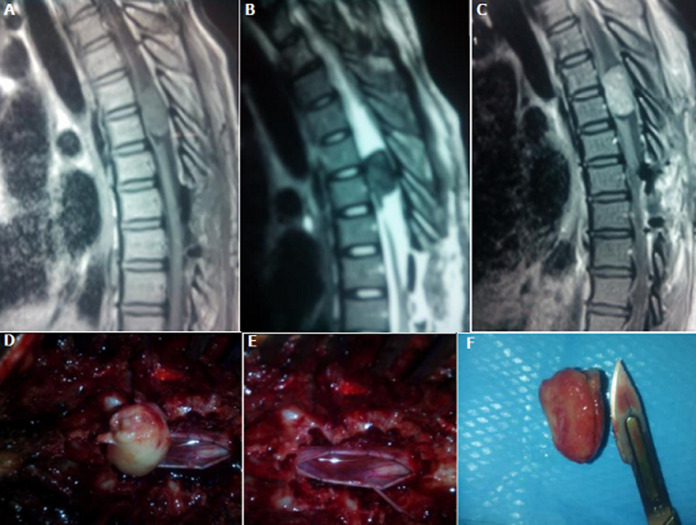
images pré et peropératoires d'un méningiome thoracique en T5-T6 avec une exérèse macroscopiquement complète

**Figure 2:**
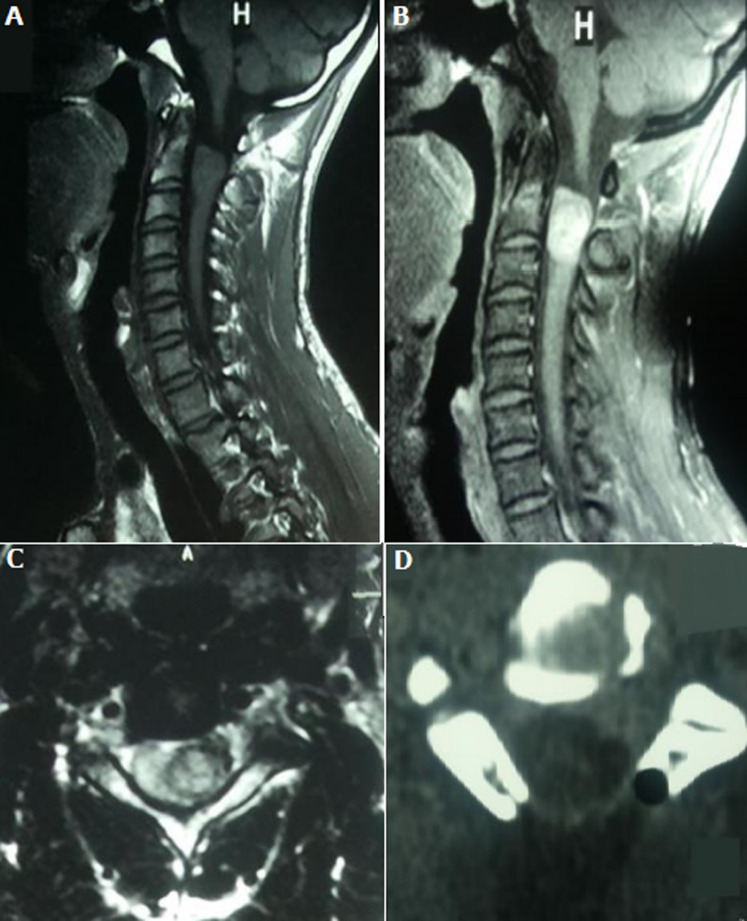
imagerie médicale en pré et postopératoire mettant en évidence une lésion faisant évoquer un neurinome en C2-C3 avec une exérèse partielle

**Figure 3:**
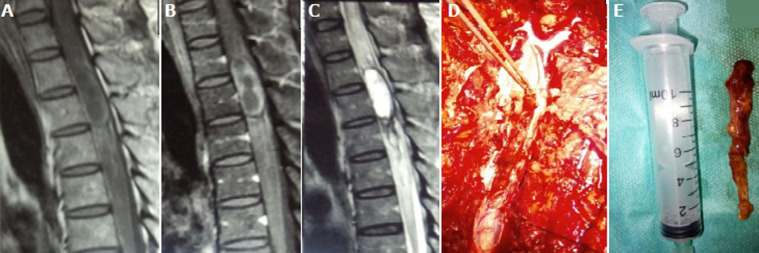
images pré et peropératoires d'une lésion intrarachidienne thoracique T1-T2-T3 pour laquelle l'anatomopathologie a conclu à un épendymome avec une exérèse macroscopiquement complète

**Données du traitement et de l´évolution**: en pré opératoire, des antalgiques de palier 1 et/ou 2 ont été prescrits à 2 patients selon l´intensité de leur douleur. En post opératoire, des antalgiques et une antibioprophylaxie ont été prescrits à tous les patients, des corticoïdes à 9 patients, des anti-inflammatoires non stéroïdiens à cinq (5) patients. Délai d´attente de l´intervention chirurgicale était de 12,4 jours avec des extrêmes de 6 et 21 jours et un écart type de 7,9. Tous les patients ont été opérés par voie postérieure. L´exérèse était macroscopiquement complète dans 14 cas, partielle dans 5 cas. Après la chirurgie, une kinésithérapie a été prescrite à tous les patients. Le cas de plasmocytome a été référé en hématologie, mais n´a pu bénéficier d´un traitement complémentaire. Les Figure 1, Figure 2 et Figure 3 représentent respectivement les images pré et peropératoires d'un méningiome thoracique avec une exérèse macroscopiquement complète; imagerie médicale en pré et postopératoire d´un neurinome cervical avec une exérèse partielle; les images pré et peropératoires d'un épendymome thoracique avec une exérèse macroscopiquement complète. La durée moyenne d´hospitalisation en post opératoire était de 4,26 jours avec des extrêmes de 3 et 6 jours et un écart type de 3,2. L´évolution post opératoire immédiate était marquée par une aggravation neurologique transitoire (aggravation ayant récupéré avant la sortie d´hôpital) dans 3 cas, un était clinique stationnaire dans 14 cas et une récupération partielles du déficit sensitif dans 2 cas. Après un recul de 6 mois de la sortie d´hôpital, 6 patients avaient récupéré totalement de tous leurs déficits neurologiques, 9 avaient récupéré partiellement et 4 étaient perdus de vue.

## Discussion

Les tumeurs médullaires sont relativement peu fréquentes. Nous avons colligé en moyenne 2,3 tumeurs par an. D´autres auteurs [7] avaient en moyenne 6,2 tumeurs par an. Le diagnostic était tardif avec un délai moyen entre le début des symptômes et la première consultation de 2,63 mois. Un long délai diagnostique avait également été noté dans plusieurs séries. Il était en moyenne de 9 mois [1]; 9,25 mois [8]; 17,83 mois [7]; 19 mois [2]. Ce long délai diagnostique pourrait expliquer le fait que la quasi-totalité des patients était au stade avancé de la souffrance neurologique et présentaient un syndrome de compression médullaire lente d´installation progressive. Les 5 patients chez qui ce syndrome de compression médullaire n´était pas encore très franc étaient parmi ceux qui avaient consulté un peu tôt. Les douleurs rachidiennes sont très fréquentes dans les tumeurs intrarachidiennes et sont souvent au premier plan au début de la maladie [7]. Elles n´ont été notées que dans 4 cas de notre série attestant encore le caractère tardif de la consultation à un moment où la douleur avait cédé probablement sous l´effet d´un traitement intempestif ou d´une automédication. Le syndrome de compression médullaire était la circonstance de diagnostic la plus fréquente dans la littérature [1,2,4]. Cependant, des auteurs [9] ont rapporté 2 cas de tumeurs (épendymome et schwannome) du cône terminal s´étendant à la queue de cheval et associé à une hydrocéphalie révélée par la triade : démence, troubles de la marche et incontinence urinaire.

A l´imagerie médicale (TDM, IRM) le siège dorsal était le plus fréquent [1,2,7]. La tumeur était extradurale dans 6 cas, intra durale dans 4 cas, intra et extradurale dans 2 cas [7]. L´IRM était l´examen de choix pour la description et l´approche histologique de ces lésions tumorales [3-6]. Dans notre série, elle était meilleure au scanner pour la description de la tumeur dans en précisant son siège (intra médullaire, intradural ou extra dural). Par contre le nombre de diagnostics histologiques évoqué par l´IRM et confirmé à l´histologie était plus important pour le scanner que pour l´IRM. Cela pourrait s´expliquer par le fait qu´à cause de son coût, l´IRM est souvent demandée dans les cas très difficiles où le scanner n´a pu faire le diagnostic différentiel entre une atteinte tumorale ou une autre atteinte (infectieuse, vasculaire). Dans ce cas la priorité de l´IRM est plus de faire ce diagnostic différentiel et de bien décrire la lésion (donner le siège et les rapports précis) que de donner un diagnostic histologique (qui sera confirmé par l´histologie). En outre aucun scanner n´est venu préciser les données d´une IRM, bien au contraire c´était l´IRM qui a précisé les données du scanner dans 2 cas. Les types histologiques les plus fréquents notés dans notre série étaient les méningiomes suivis des neurinomes puis des épendymes. Dans d´autres études [2,4], les épendymome étaient les plus fréquents. Plusieurs autres types histologiques rares pour la plupart ont été rapportés le plus souvent sous forme d´un cas clinique dans la littérature [2,6,7,10-14] : neuroblastome, lipome intra médullaire non dysgraphique, neurofibromes, neuro fibrosarcome, sacome granulocytaire, métastase d´une tumeur colique, plasmocytome, myélome multiple, angiolipome, hemagioblastome, astrocytome.

Sur le plan thérapeutique ; la chirurgie est presque toujours nécessaire pour confirmer le diagnostic et pour le traitement [15]. L´exérèse macroscopiquement complète est le traitement le plus efficace dans le cas des tumeurs médullaires bénignes [7]. La biopsie chirurgicale joue également un rôle important pour l´obtention du diagnostic histologique dans les autres cas, car une chimiothérapie et/ou radiothérapie peuvent être indiquées [10,16]. L´exérèse était macroscopiquement complète dans 63,7% des cas, incomplète dans 18,1% des cas et biopsique dans 3% dans cas [15]. Sur 12 cas, l´exérèse était macroscopiquement complète dans neuf cas, partielle dans trois cas [7]. Elle était totale dans 5 cas, partielle dans 4 cas et biopsique dans 1 cas [2]. Nous n´avions aucun cas de biopsie, car celle-ci ne faisait pas partie de nos indications chirurgicales étant donné qu´aucun traitement complémentaire (radio ou chimiothérapie n´était disponible dans notre pays). Ainsi aucun patient de notre série n´a bénéficié d´un traitement complémentaire même lorsqu´il y avait une indication (cas du plasmocytome). Plusieurs auteurs [10,14,17,18] avaient réalisé une chimiothérapie anticancéreuse dans les cas de myélome multiple, de plasmocytome, de sarcome granulocytaire, de neuroblastome. D´autres auteurs [10,19] pratiquaient une radiothérapie dans les cas de plasmocytome solitaire osseux et de neurofibrosarcome primitif. En post opératoire, Il n´y a pas eu de complication infectieuse ni de décès jusqu´à la sortie des patients. De même sur le plan neurologique les suites étaient favorables, car les quelques cas d´aggravation neurologiques post opératoire ont rapidement retrouvé leur état neurologique antérieur (pré opératoire) avant leur sortie d´hôpital. A moyen ou long terme, nos résultats étaient toujours satisfaisants, car la totalité des patients revus avait récupéré soit totalement soit partiellement. Ces résultats satisfaisants à long terme dans notre série pourraient s´expliquer par le fait que le manque de traitement complémentaire nous amène le plus souvent à n´opérer que les tumeurs d´allure bénignes à l´imagerie. D´autres auteurs [1] notaient 61,54% de récupération motrice partielle. Une récupération totale avait été observée dans un cas de neuroblastome [14]. Cette évolution favorable des tumeurs intra rachidiennes après un traitement adéquat a été notée part la grande majorité des auteurs [6,7,9,10,13]. Certaines complications ont cependant été notées. Ainsi, sur 10 cas il y avait 3 aggravations neurologiques post opératoires, et 2 décès par complication de décubitus [2]. Ailleurs, sur 3 cas, il y avait 1 cas d´embolie pulmonaire [10]. Des auteures [1] ont conclu que l´évolution dépend du stade neurologique initial.

## Conclusion

Les tumeurs intrarachidiennes étaient relativement peu fréquentes dans notre étude et prédominaient chez l´adulte de sexe masculin. Le diagnostic était tardif et toujours au stade de déficit neurologique. L´IRM a été l´imagerie médicale la plus réalisée pour le diagnostic positif des tumeurs intrarachidiennes. Elle était supérieure à la TDM pour la description précise (siège, rapports) de la tumeur. Toutefois l´IRM gardait quelques limites dans les approches histologiques. La concordance radio histologique était meilleure pour méningiomes et les neurinomes aussi bien au scanner qu´à l´IRM. Le méningiome était de loin le type histologique le plus fréquent. L´exérèse chirurgicale était macroscopiquement complète dans la majorité des cas. Les suites opératoires étaient simples. L´évolution neurologique était favorable dans la majorité des cas.

### Etat des connaissances sur le sujet

- Les aspects diagnostiques;
- Les aspects thérapeutiques.

### Contribution de notre étude à la connaissance

- Les difficultés diagnostiques dans notre contexte de travail;
- Le profil histologique des patients opérés dans notre service;
- Le traitement réalisé dans notre pays et ses résultats.
